# Supplementary material for: Saturation genome editing of DDX3X clarifies pathogenicity of germline and somatic variation
Source: Nat Commun. 2023 Dec 6;14:7702. doi: 10.1038/s41467-023-43041-4 (PMC10700591; doi:10.1038/s41467-023-43041-4)
Supplement: Supplementary file 1 — Supplementary Information [file 41467_2023_43041_MOESM1_ESM.pdf]

Supplementary Materials

1) **Supplementary Figures**
   - Fig. S1: Correlation between sgRNAs and between measures of variant abundance
   - Fig. S2: Additional characterisation of SGE-depleted and SGE-enriched variants.
   - Fig. S3: Map of all SNVs and codon deletion variants' SGE functional class
   - Fig S4: cLFC trend of all DDX3X variants, grouped by exon
   - Fig S5: cLFC trend of all DDX3X variants, grouped by variant type
   - Fig S6: Map of all SNVs and codon deletion variants' predicted relevance for neurodevelopmental disorders
   - Fig S7: Distribution of classifier of NDD-relevant functional abnormality posterior probabilities
   - Fig S8: dNdScv analysis of individual cancer types
   - Fig. S9: Characterization of the HAP1 cell bank
   - Fig. S10: Optimisation of Saturation Genome Editing
   - Fig. S11: Correlation between cLFC trend and average redundant codon cLFC trend

2) **Supplementary Results**
   - Optimization of the SGE methodology
   - Genotype-phenotype correlation
   - Discrepancies between SGE Data and Functional Data

3) **Supplementary References**

Supplementary Figures

Fig. S1

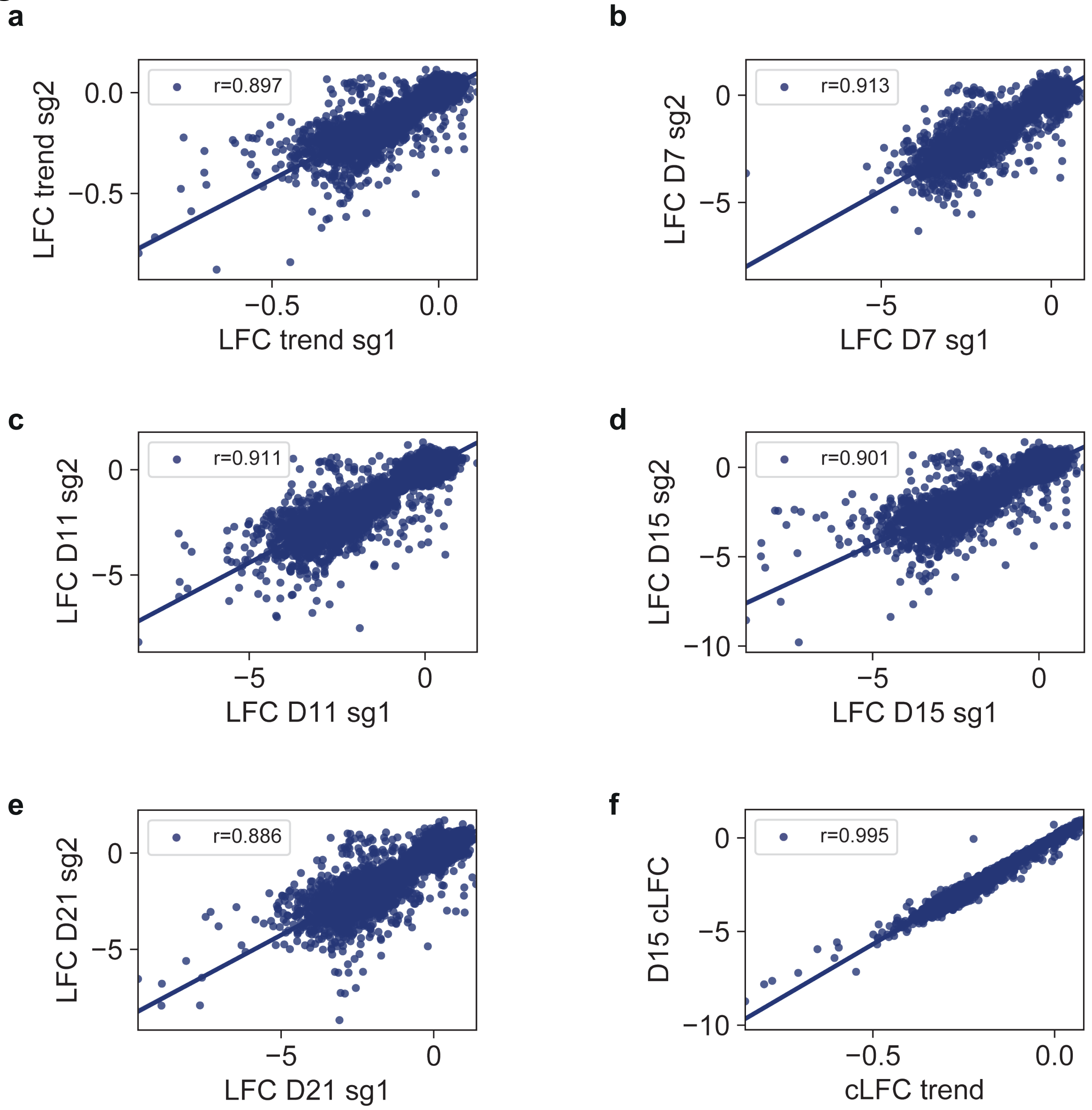

Fig. S1. Correlation between sgRNAs and between measures of variant abundance. a) Correlation between LFC-trend for sg1 and sg2; Pearson r=0.897. b) Correlation between Day 7 LFC for sg1 and sg2; Pearson r=0.913. c) Correlation between Day 11 LFC for sg1 and sg2; Pearson r=0.911. d) Correlation between Day 15 LFC for sg1 and sg2; Pearson r=0.901. e) Correlation between Day 21 LFC for sg1 and sg2; Pearson r=0.886. f) Correlation between Day 15 cLFC and cLFC trend; Pearson r=0.995. Source data are provided as a Source Data file.

Fig. S2

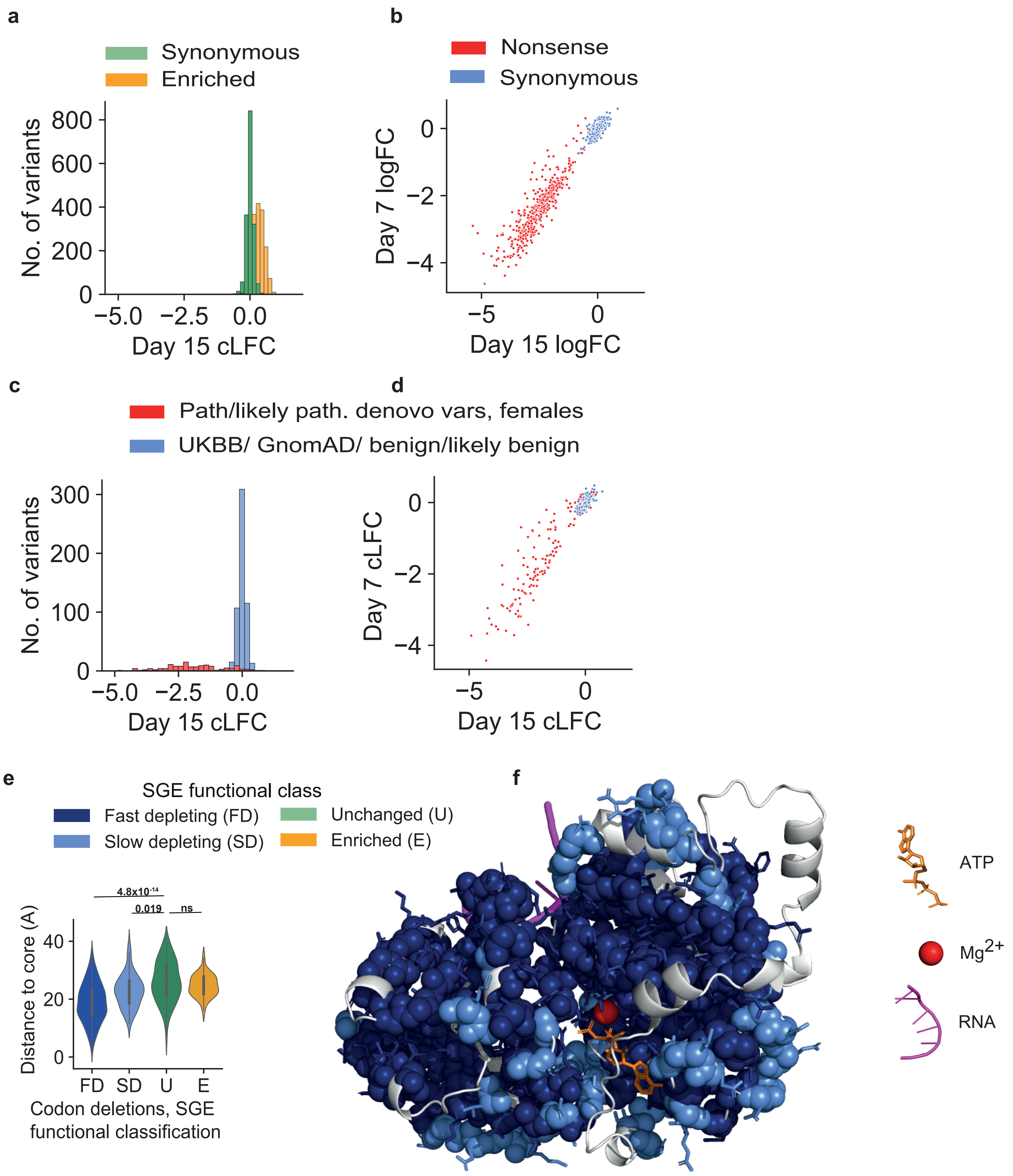
a
Synonymous
Enriched
No. of variants
800
600
400
200
0
−5.0
−2.5
0.0
Day 15 cLFC
b
Nonsense
Synonymous
Day 7 logFC
0
−2
−4
−5
0
Day 15 logFC
c
Path/likely path. denovo vars, females
UKBB/ GnomAD/ benign/likely benign
No. of variants
300
200
100
0
−5.0
−2.5
0.0
Day 15 cLFC
d
Day 7 cLFC
0
−2
−4
−5
0
Day 15 cLFC
e
SGE functional class
Fast depleting (FD)
Slow depleting (SD)
Unchanged (U)
Enriched (E)
4.8x10-14
0.019
ns
Distance to core (A)
40
20
0
FD
SD
U
E
Codon deletions, SGE functional classification
f
ATP
Mg2+
RNA

Fig S2: Further characterisation of SGE-depleted and SGE-enriched variants.
a) Day 15 cLFC of variant abundance of synonymous and SGE-enriched variants. b) Day 7 and Day 15 cLFC of nonsense and synonymous variants. c&d) red: de novo pathogenic/likely pathogenic variants observed in female patients, blue: benign/likely benign variants and variants observed in GnomAD and UKBB. c) Day 15 cLFC. d) Day 7 and Day 15 cLFC. e) Distance from the centroid of DDX3X to the amino-acid side chain centroid (Ångstrom), codon-deletion variants, KW statistic = 75, p=3.3x10-16. Dunn's BH-corrected FDR. FD: Fast-depleting n=233, SD: Slow-depleting n=71, U: SGE-unchanged n=88, E: SGE-enriched n=31, internal boxplots within the violinplot show median and interquartile range (IQR), whiskers denote 1.5xIQR. f) Alphafold2 DDX3X structure together with ATP, magnesium ion and RNA. Coloured according to the SGE functional class for residue deletion. Spheres: residue main chain. Sticks: residue side chain. Source data are provided as a Source Data file.

Fig. S3

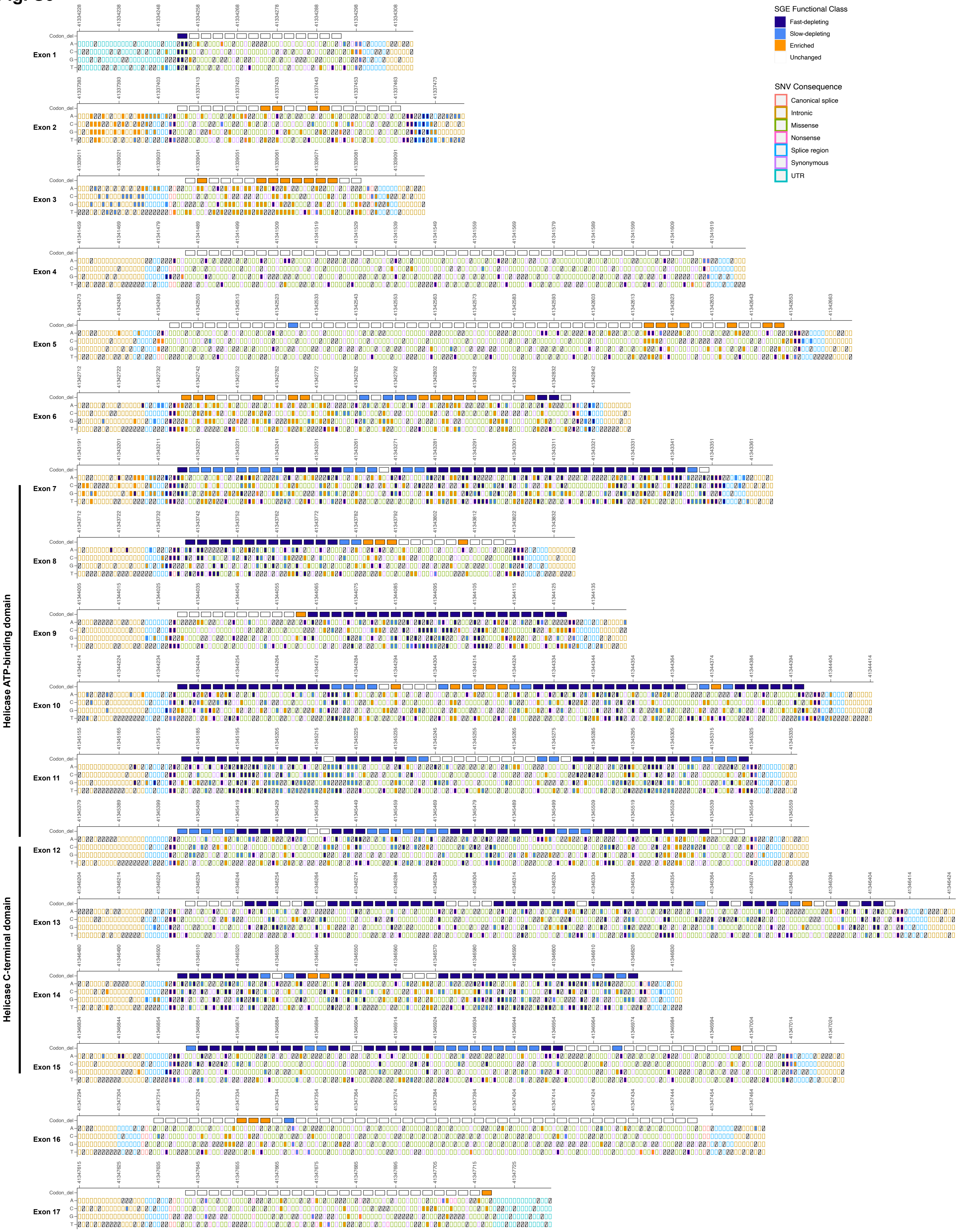
SGE Functional Class
Fast-depleting
Slow-depleting
Enriched
Unchanged
SNV Consequence
Canonical splice
Intronic
Missense
Nonsense
Splice region
Synonymous
UTR
Codon_del
A
C
G
T
Exon 1
Exon 2
Exon 3
Exon 4
Exon 5
Exon 6
Exon 7
Exon 8
Exon 9
Exon 10
Exon 11
Exon 12
Exon 13
Exon 14
Exon 15
Exon 16
Exon 17
Helicase ATP-binding domain
Helicase C-terminal domain

Fig S3: Map of all SNVs and codon deletion variants' SGE functional class
This figure displays the SGE functional class of all 7,944 nucleotide (SNVs) and 626 codon deletion (Codon_del) variants in DDX3X. The x-axis of each sub-panel shows the chromosome X coordinate based on the hg38 reference genome. The outline colour of each box represents the predicted functional consequence of the SNV, while the fill colour reflects the SGE functional class. Source data are provided as a Source Data file.

Fig. S4

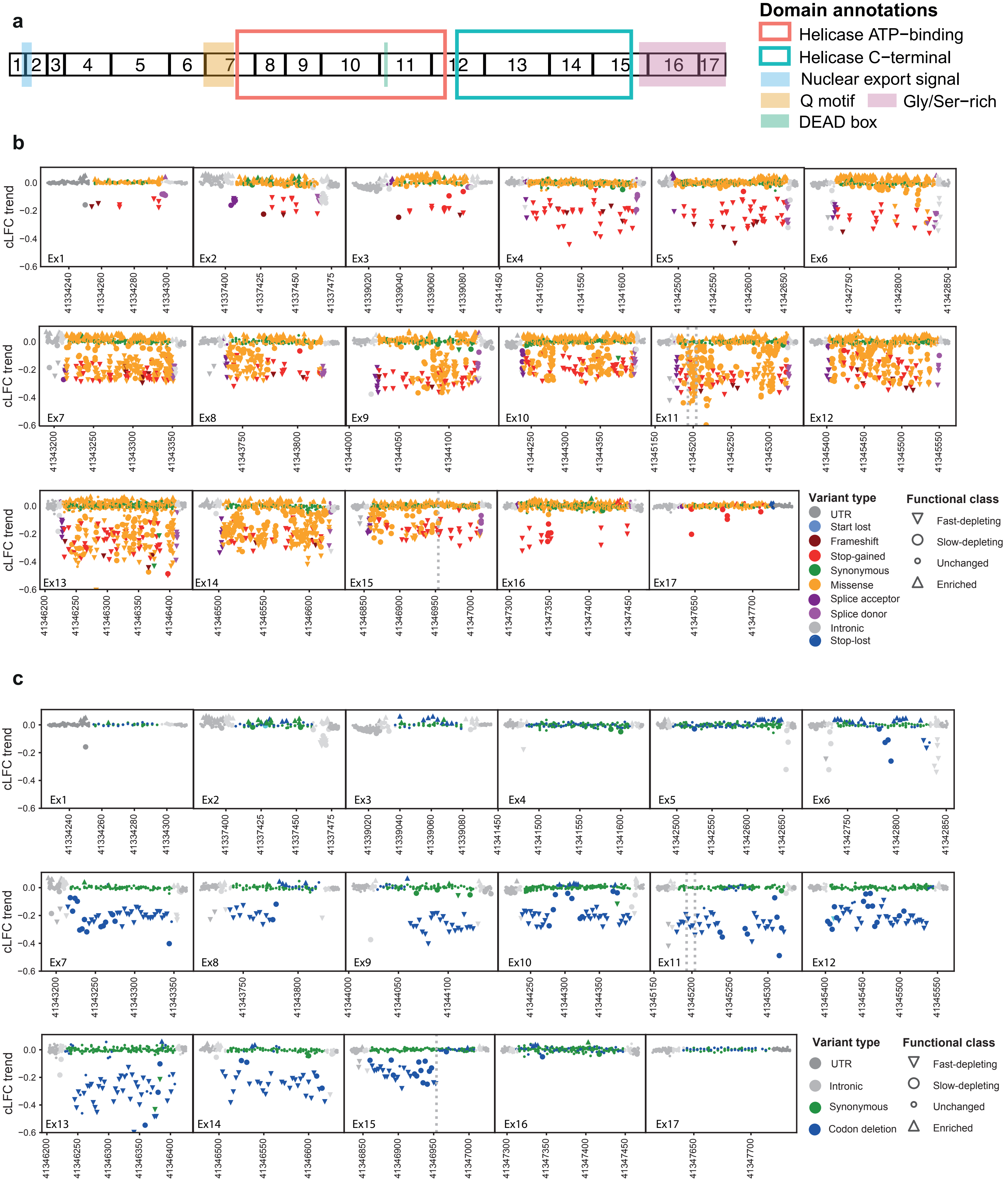

Fig. S4. cLFC trend of all DDX3X variants, grouped by exon
a) Top panel: DDX3X exon structure with locations of key domains and protein annotations. b and c) y-axis: cLFC-trend; x-axis: Chromosome X hg38 position, grouped by exon. Variants coloured by variant type, shape indicates SGE functional class. Codon deletion variants are shown separately in c for clarity.
Source data are provided as a Source Data file.

Fig. S5

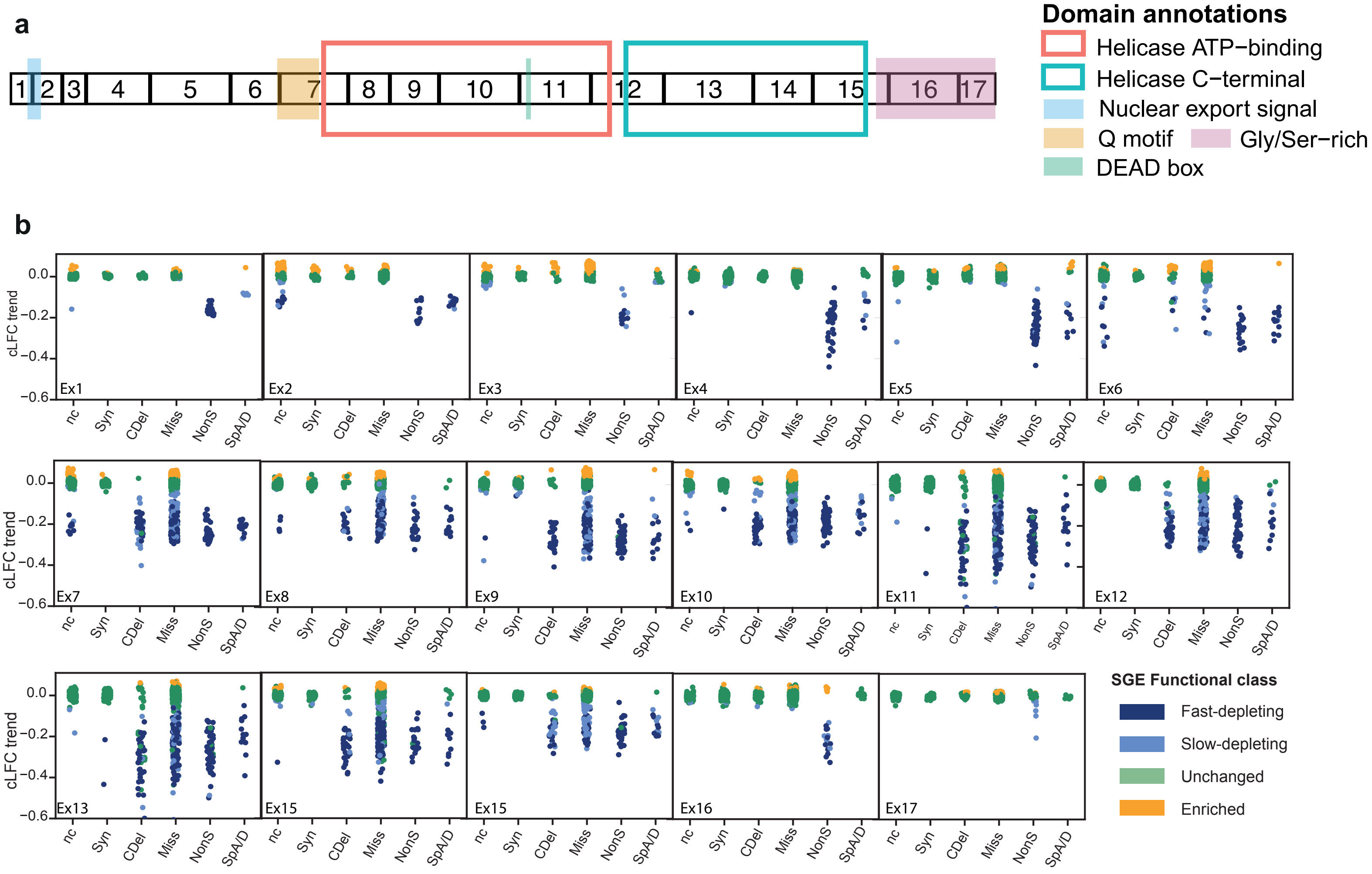

Fig. S5. cLFC trend of all DDX3X variants, grouped by variant type
a) DDX3X exon structure with locations of key domains and protein annotations. b) cLFC-trend of non-coding (nc), synonymous (Syn), in-frame codon-deletion (Cdel), missense (Miss), nonsense (NonS) and canonical splice acceptor/donor variants (SpA/D), grouped by exon and coloured by SGE functional class. Source data are provided as a Source Data file.

Fig. S6

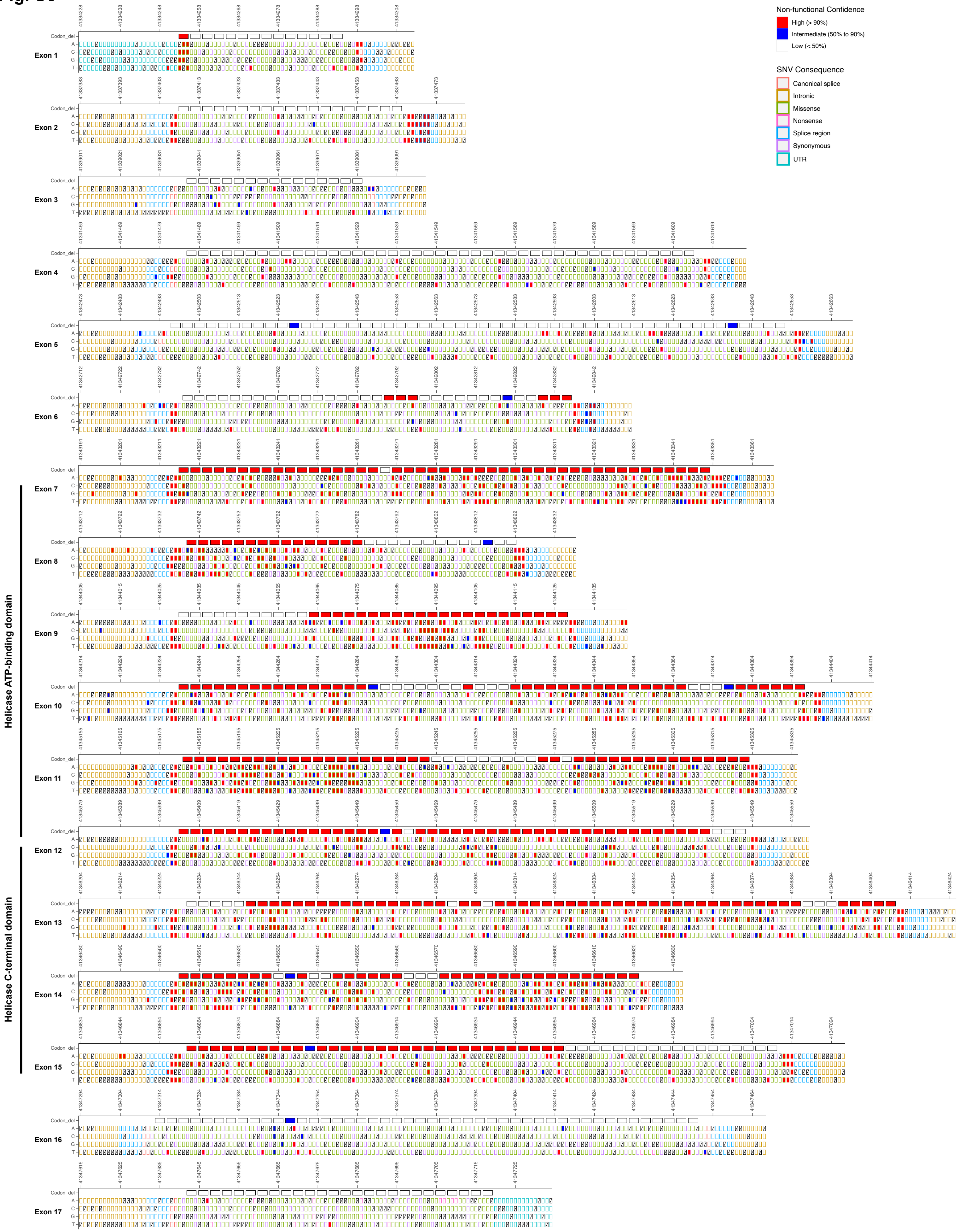
Non-functional Confidence
High (> 90%)
Intermediate (50% to 90%)
Low (< 50%)
SNV Consequence
Canonical splice
Intronic
Missense
Nonsense
Splice region
Synonymous
UTR
Codon_del
A
C
G
T
Exon 1
Exon 2
Exon 3
Exon 4
Exon 5
Exon 6
Exon 7
Exon 8
Exon 9
Exon 10
Exon 11
Exon 12
Exon 13
Exon 14
Exon 15
Exon 16
Exon 17
Helicase ATP-binding domain
Helicase C-terminal domain

Fig. S6: Sequence-function map of *DDX3X*

This figure displays the relevance to *DDX3X*-related neurodevelopmental disorder for all 7,944 nucleotide (SNVs) and 626 codon deletion (Codon_del) variants in DDX3X. The x-axis of each sub-panel shows the chromosome X coordinate based on the hg38 reference genome. The outline colour of each box represents the predicted functional impact of the SNV, while the fill colour reflects the degree of confidence that a variant results in a non-functional protein, calculated using the random forest model. Source data are provided as a Source Data file.

Fig. S7

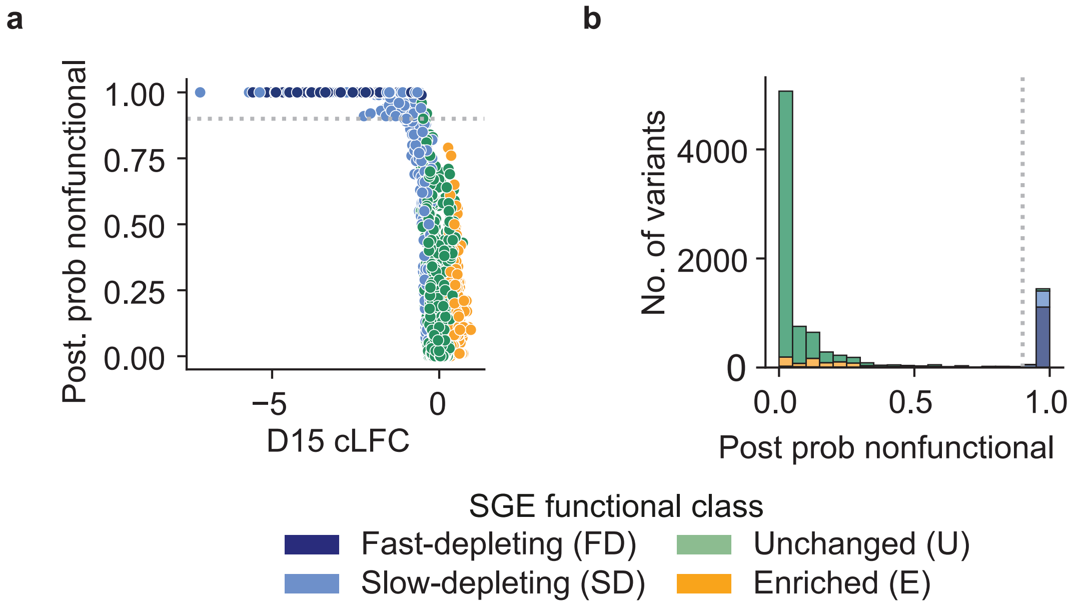

Fig. S7: Distribution of classifier of NDD-relevant functional abnormality posterior probabilities

a) Posterior probability that a variant results in a functionally abnormal protein from the Random Forest NDD-relevance classifier (y-axis) plotted against the Day 15 cLFC. Fast and slow depleting variants cluster above a posterior probability threshold of 0.9 (grey dotted line). b) Distribution of variants' Random Forest classifier posterior probability. Grey dotted line posterior probability = 0.9. Source data are provided as a Source Data file.

Fig. S8

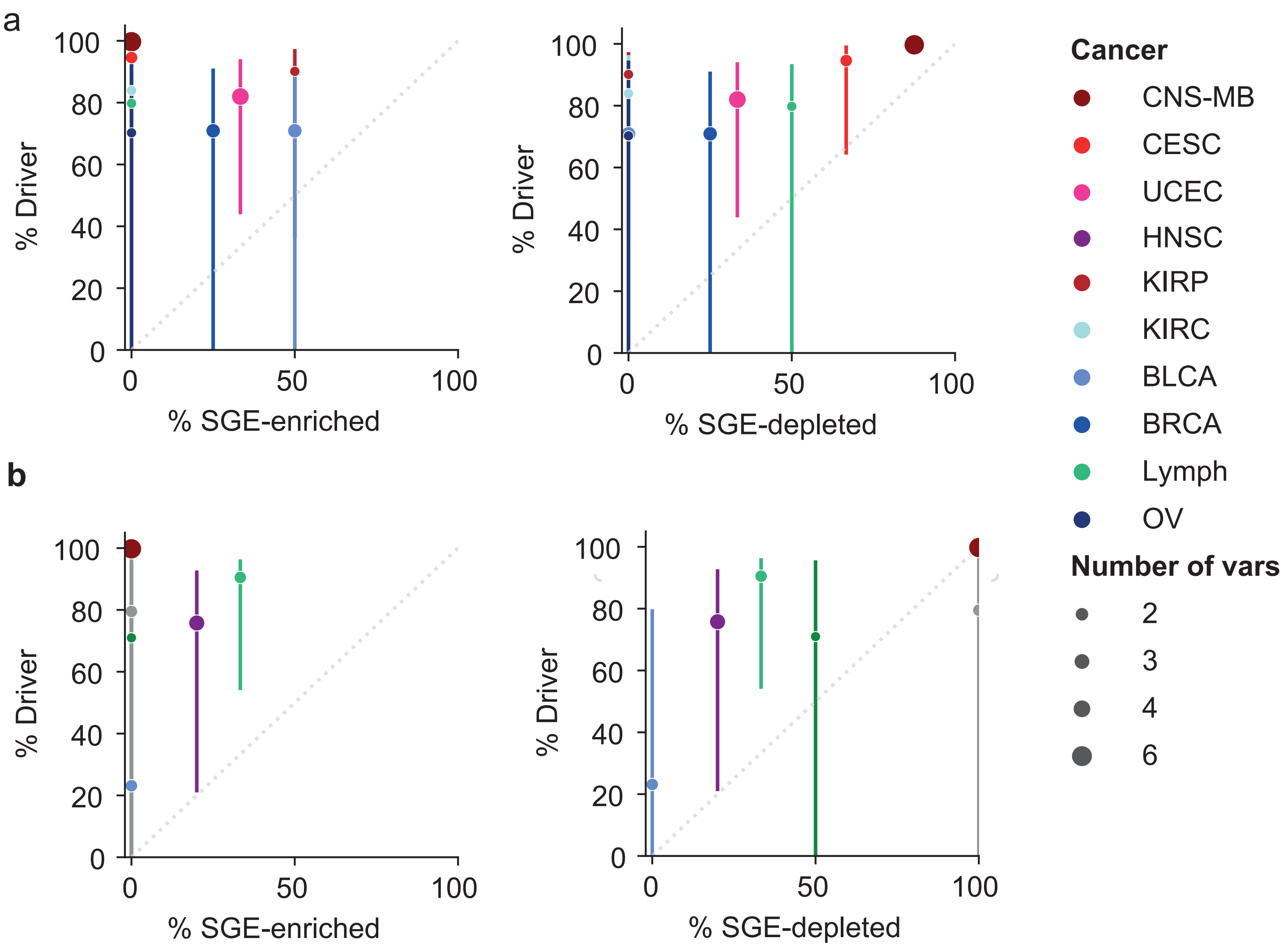

Fig. S8: dNdScv analysis of individual cancer types

The proportion of missense variants classified as SGE-enriched and SGE-depleted (x-axis), and the percentage of missense variants that are likely drivers (y-axis), estimated from the observed:expected number of DDX3X missense variants (dN/dS), in different cancer types where there is more than 1 missense DDX3X variant in A) females and B) males. Error bars denote 95% CI. CNS-MB: Central nervous system medulloblastoma; CESC: Cervical squamous cell carcinoma and endocervical adenocarcinoma; UCEC: Uterine Corpus Endometrial Carcinoma; HNSC: Head and Neck squamous cell carcinoma; KIRP : Kidney renal papillary cell carcinoma; KIRC : Kidney renal clear cell carcinoma; BLCA: Bladder Urothelial carcinoma; BRCA: Breast invasive carcinoma; Lymph: Lymphomas; OV: Ovarian serous cystadenocarcinoma. Error bars show 95% confidence intervals. Source data are provided as a Source Data file.

Fig. S9

a

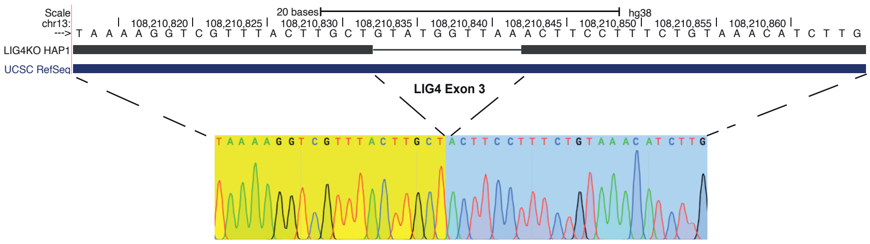

b

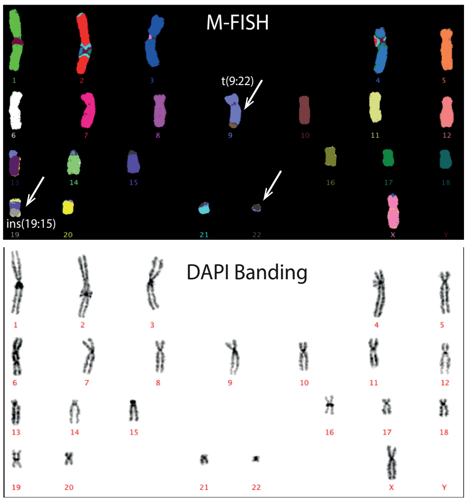

Fig. S9. Characterization of the HAP1 cell bank

a) Sanger sequencing shows 10bp deletion within exon 3 of the LIG4 gene. b) Representative karyotyping by M-FISH and DAPI banding showing haploid cells with the expected t(9;22) and ins(15;19) karyotype.

Fig. S10

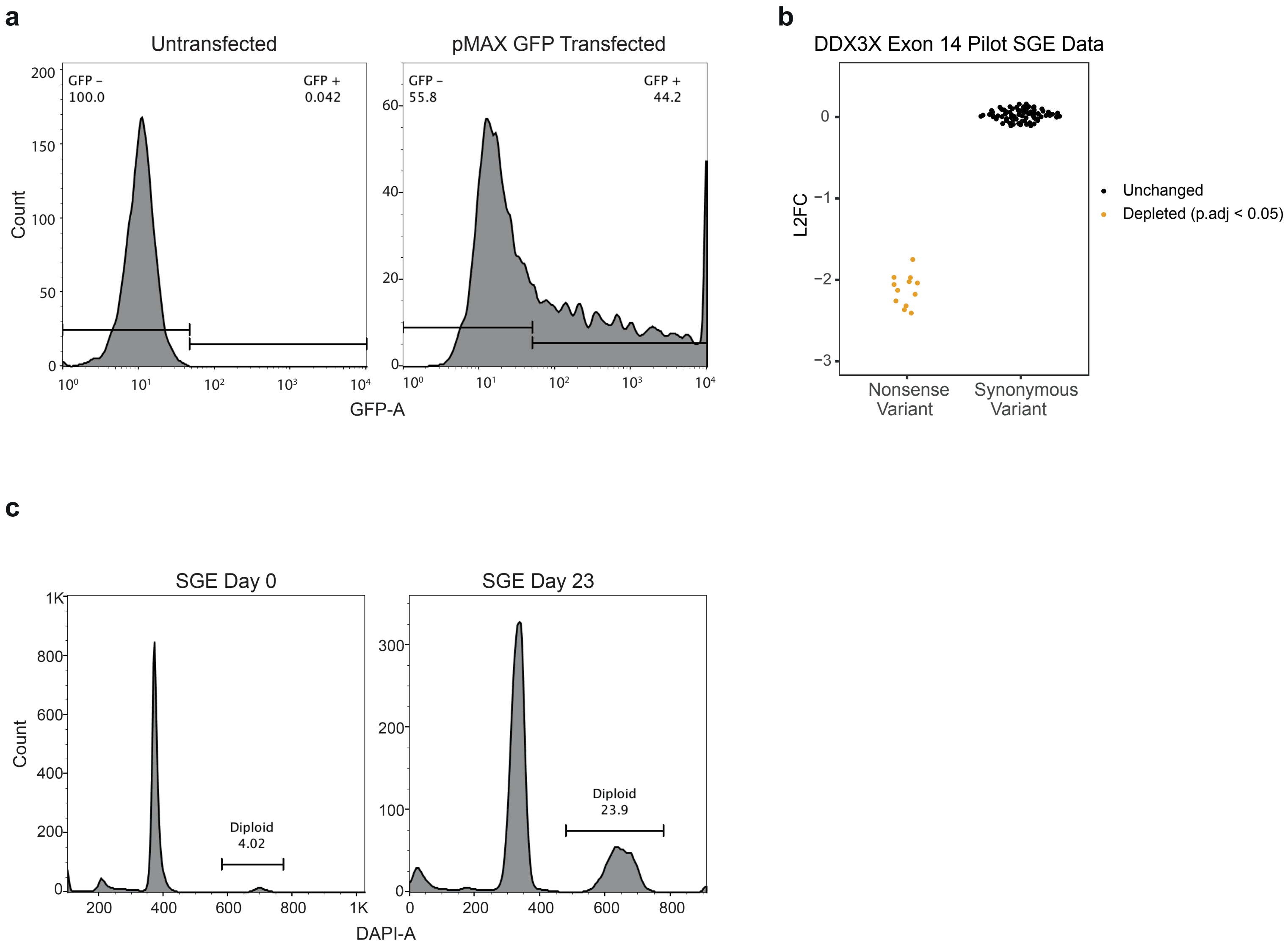

Fig. S10. Optimisation of Saturation Genome Editing

a) Estimation of co-transfection efficiency of the SGE protocol. pMin-U6-DDX3XsgRNA-hPGK-puro and pMAX GFP plasmid were used for the co-transfection. Representative FACS plot showing 44% co-transfection efficiency. Only the viable singlet cells were used for the analysis. b) Pilot SGE study of *DDX3X* exon 14. Day 11 vs Day 4 Log2 fold-change of nonsense and synonymous variants. Depleted variants are indicated by a Benjamini-Hochberg (BH) corrected False Discovery Rate (FDR) < 0.05.
c) On Day 0 of a representative SGE experiment, the percentage of diploid cells was 4.07%. On Day 23 of a representative SGE experiment, the percentage of diploid cells was 23.9%. Only the singlet cells were used for the analysis.

Fig. S11

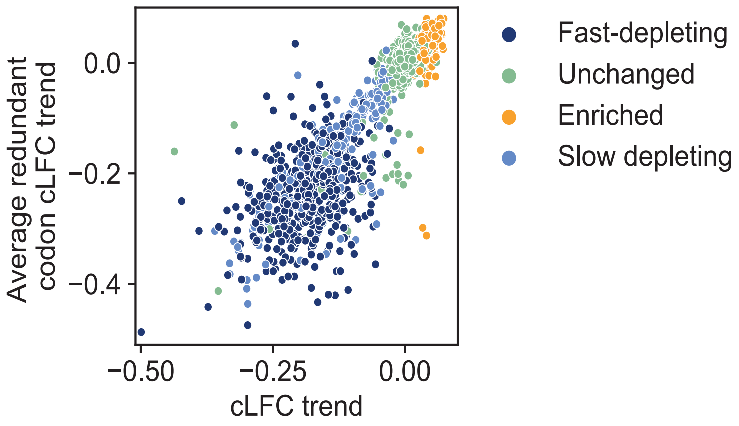

Fig. S11. Correlation between cLFC trend and average redundant codon cLFC trend
95% of SGE-depleted variants had a redundant codon which was classified as SGE-depleted. 69% of SGE-enriched variants had a redundant codon which was classified as SGE-enriched. The cLFC trend of redundant codons have directional consistencies of 99% and 95% with SGE-depleted and SGE-enriched variants, respectively. Source data are provided as a Source Data file.

Supplementary Results

Optimization of the SGE methodology

We made several refinements to improve the sensitivity in the SGE assay described in Findlay *et. al.*[1]. Most notably, we increased the transfection efficiency in HAP1 cells to 40-50% (Fig. S10a) by i) reducing the size of the transfected plasmids, ii) integrating the large Cas9 expressing construct into the cell prior to SGE and iii) re-optimizing the transfection conditions. High transfection efficiencies should increase the representation of the tested variants, improving the signal-to-noise ratio in our SGE assay. In a pilot SGE study of *DDX3X*, we observed a strong depletion of all nonsense variants relative to synonymous variants on Day 11 in exon 14, confirming that *DDX3X* is essential in HAP1 cells. It also suggested that our adapted SGE protocol generates data with a high signal-to-noise ratio (Fig. S10b).

We also observed that the FACS-sorted haploid HAP1 cells could preserve their high haploidy after one to two weeks of expansion with one freeze-thaw cycle. This two-week culture window allows us to prepare a haploid HAP1 cell bank for all the SGE experiments performed in this study. Using the same source and passage number of HAP1 cells could potentially reduce undesired biological batch effects and improve the consistency and sensitivity of our SGE assay. The diploid population of this HAP1 cell bank increased from 4% to 24% after 23 days of the SGE experiment, suggesting that the time-course experiment should not go beyond Week 3 (Fig. S10c). In order to capture the different kinetics of variant effects, we harvested the cell pellet at 5 timepoints of the 3-week experiment. Results from multiple timepoints allowed us to identify loss-of-function variants with different kinetics of variant effect (fast- and slow-depleting) and a subset of *DDX3X* variants (enriched) which do not appear to act through a loss-of-function mechanism. However, these enriched variants are rarely observed in healthy individuals. During the SGE data analysis, Day 15 cLFC was used in preference to Day 21 cLFC as a subtle increase in mean variant abundance was observed from Day 15 to Day 21, likely due to increased diploidy within the assayed cell population at this later time point.

In the design of the variant libraries, we introduced a class of multiple nucleotide variants (MNVs), which we refer to as redundant codons. The redundant codon details were described in

the ‘snvre’ option of our VaLiAnT package, a tool used for SGE oligo design[2]. In short, we introduced an alternative codon for each designed non-synonymous SNV, and all possible synonymous codons for each amino acid. We maximised the number of synonymous variants in the library because we heavily rely on the synonymous variants to establish the LFC/LFC-trend baseline for normalisation during the data analysis. These redundant synonymous variants were particularly important for short exons during LFC/LFC-trend normalisation, such as Exon 3 of *DDX3X*. Moreover, the redundant codons confirm the concordance of the variant effect at the amino acid level. In our data, we observed that both SGE-depleted and SGE-enriched variants exhibited good concordance between redundant variants: 95% of SGE-depleted variants had a redundant codon that was also classified as SGE-depleted, while 69% of SGE-enriched variants had a redundant codon that was also classified as SGE-enriched, with directional consistencies of 99% and 95%, respectively (Fig. S11).

Genotype-phenotype correlation

To investigate whether there are differences in the severity of intellectual disability between *DDX3X*-related NDD probands who carry SGE-fast and SGE-slow-depleting variants we identified 61 probands who had undergone assessment via the Vineland Adaptive Behaviour Scales[3,4] across three studies[5–7]. No difference in global adaptive function (Vineland Adaptive Behaviour Composite score) was observed between individuals carrying missense variants and those carrying protein-truncating variants (PTVs) (Fig.5b). There was also no significant difference in the global adaptive function of individuals carrying fast or slow-depleting *DDX3X* variants (two-tailed t-test p=0.27, Fig. 5c). To investigate phenotypes more broadly, a composite score was devised for the Lennox *et al.* cohort, encompassing brain MRI findings, microcephaly, sensory deficits, muscle tone anomalies, cardiac findings, precocious puberty, experience of seizures and behavioural assessment. We observed no significant difference in composite score between fast and slow-depleting variants (Fig.5c). Finally, we observed no difference in the rate of attainment of developmental milestones (age of speaking first words or taking first independent steps) in patients in the DDD cohort carrying fast or slow-depleting variants (Fig.5e,f).

Discrepancies between SGE Data and Functional Data

Relatively few *DDX3X* variants have been functionally characterised previously, against which we could compare our results. Fonseca *et. al.* proposed that the L556S and R376C *de novo* variants observed in female patients render DDX3X prone to protein aggregation[8]. Both variants are functionally abnormal depleting variants in our assay. Kellaris *et al.* modelled *in vivo* in zebrafish embryos the R79K variant seen in two male siblings with NDD and proposed that this variant results in a partial loss-of-function[9]. This variant is functionally normal in our assay. Snijders-Blok *et al.*[5–7,10] applied the same *in vivo* assay to assess 3 variants observed in male probands and 5 observed in females and observed all male variants to be no different to wild-type. Of the 5 female variants for which they identified loss-of-function effects (I214T, R326H, R376C, I507T, R534H), 4 are functionally abnormal depleting variants in our assay. Of the 8 variants previously determined to have negatively impacted helicase activity[6], 7 are functionally abnormal depleting variants in our assay. The remaining variant, R326H, was interpreted to be pathogenic and functionally abnormal by both Lennox and Snijders Blok *et al.*. The SNV generating R326H in our assay is classified as functionally normal. However, the multi-nucleotide redundant variant for the same amino-acid change is a functionally abnormal, fast-depleting variant.

Supplementary References

1. Findlay, G. M. *et al.* Accurate classification of BRCA1 variants with saturation genome editing. *Nature* **562**, 217–222 (2018).
2. Barbon, L. *et al.* Variant Library Annotation Tool (VaLiAnT): an oligonucleotide library design and annotation tool for Saturation Genome Editing and other Deep Mutational Scanning experiments. *Bioinformatics* (2021) doi:10.1093/bioinformatics/btab776.
3. *Vineland adaptive behavior scales Vineland-II: Survey forms manual*. (2005).
4. Pepperdine, C. R. & McCrimmon, A. W. Test review: Vineland adaptive behavior scales, third edition (Vineland-3) by sparrow, S. s., Cicchetti, D. v., & Saulnier, C. a. *Can. J. Sch. Psychol.* **33**, 157–163 (2018).
5. Tang, L. *et al.* Prospective and detailed behavioral phenotyping in DDX3X syndrome. *Mol. Autism* **12**, 36 (2021).
6. Lennox, A. L. *et al.* Pathogenic DDX3X mutations impair RNA metabolism and neurogenesis during fetal cortical development. *Neuron* **106**, 404-420.e8 (2020).
7. Ng-Cordell, E. *et al.* Social and emotional characteristics of girls and young women with DDX3X-associated intellectual disability: a descriptive and comparative study. *J. Autism Dev. Disord.* **53**, 3208–3219 (2023).
8. de Castro Fonseca, M. *et al.* Molecular and cellular basis of hyperassembly and protein aggregation driven by a rare pathogenic mutation in DDX3X. *iScience* **24**, 102841 (2021).
9. Kellaris, G. *et al.* A hypomorphic inherited pathogenic variant in DDX3X causes male intellectual disability with additional neurodevelopmental and neurodegenerative features. *Hum. Genomics* **12**, (2018).

10. Snijders Blok, L. *et al.* Mutations in DDX3X are a common cause of unexplained intellectual disability with gender-specific effects on wnt signaling. *Am. J. Hum. Genet.* **97**, 343–352 (2015).
