# Supplementary material for: Saturation genome editing of DDX3X clarifies pathogenicity of germline and somatic variation
Source: Nat Commun. 2023 Dec 6;14:7702. doi: 10.1038/s41467-023-43041-4 (PMC10700591; doi:10.1038/s41467-023-43041-4)
Supplement: Supplementary file 3 — Description of Additional Supplementary Files [file 41467_2023_43041_MOESM3_ESM.pdf]

Description of Supplementary Data and Movies

Supplementary Data Legends:

Supplementary Data S1: PheWAS results of patients carrying SGE-enriched *DDX3X* variants in the UKBB.
Supplementary Data S2: Performance of different types of supervised machine learning classifiers for identifying variants relevant for NDD.
Supplementary Data S3: Performance of *in silico* variant effect prediction algorithms using default developer-recommended thresholds (see methods for details), and comparison to SGE data.
Supplementary Data S4: Modelled impact of utilisation of SGE data according to Brnich *et al.* 2019 guidelines on the utilisation of functional data in clinical variant classification. Variants are modelled as if they were observed *de novo.*
Supplementary Data S5: Modelled impact of utilisation of SGE data according to Brnich *et al.* 2019 guidelines on the utilisation of functional data in clinical variant classification. Modelled variants are of unknown inheritance.
Supplementary Data S6: Prediction of variants' functional relevance for DDX3X-related neurodevelopmental disorder, for all of 12,776 *DDX3X* variants tested, based on a Random-Forest supervised classifier, together with the confidence with which a variant is predicted to be functionally abnormal. *These are the metrics that we recommend to be incorporated into clinical variant classification.*
Supplementary Data S7: Oligonucleotide sequences synthesised for use with sgRNA1 for each exon
Supplementary Data S7: Oligonucleotide sequences synthesised for use with sgRNA2 for each exon
Supplementary Data S9: Sequence for primers used for TWIST oligo amplification.
Supplementary Data S10: sequence of pMin-U6-ccdb-hPGK-puro, used for both sgRNA plasmid and HDR template library plasmid constructs
Supplementary Data S11: Sequence of all sgRNAs used in this study.
Supplementary Data S12: sequence of primers used for cloning HDR libraries.
Supplementary Data S13: Primers and PCR cycling conditions used for each exon's sequencing library preparation.
Supplementary Data S14: SGE data for all of 12,776 DDX3X variants tested. Annotations include variant consequence, SGE functional classification, cLFC, cLFC-trend values and BH-adjusted FDR.
Supplementary Data S15: 200 pairwise alignments with a minimum identity of 25% selected from BLASTP run on the protein sequence of human DDX3X (UniProt ID O00571) against the UniRef90 database with the following parameters: *--matrix BLOSUM62 --exp 10 --dropoff 0 --alignments 1000 --scores 1000 --gapopen 10 --gapext 1 --align 0 --filter F --async*
Supplementary Data S16: DDX3X Scorecons conservation scores, and the amino acid variation at each residue position.

Supplementary Data S17: Clinical variants reported in the context of *DDX3X*-related neurodevelopmental syndrome were identified from the following sources: ClinVar (4th December 2020); DECIPHER (4th December 2020); Genomics England 100,000 genomes study (21st January 2021);

the DDD project (4th December 2020), Lennox *et al.* 2020 and Kaplanis and Samocha *et al.* 2020. NB an individual patient may have been reported by multiple sources.

Supplementary Data S18: The germline mutational probability for each SNV in *DDX3X* as per the triplet-based neutral mutational model (Samocha *et al.* 2014).

Supplementary Data S19: Vineland adaptive behaviour test scores for patients with DDX3X-related neurodevelopmental disorder, taken from NcCordell *et al.* 2023, Lennox *et. al.* 2020 and Tang *et. al.*, 2021).

Supplementary Data S20: Description of how Lennox *et al.* 2020 phenotyping data were converted into a numeric score.

Supplementary Data S21: Numeric phenotypic scores for each patient described by Lennox *et al.* 2020. For explanation of how the scores were derived, please see Supplementary Data 16.

Supplementary Data S22: Age (within a 6 month window) where individuals within the DDD project with a *de novo* DDX3X variant took their first independent steps.

Supplementary Data S23: Age (within a 6 month window) where individuals within the DDD project with a *de novo* DDX3X variant spoke their first words.

Supplementary Data S24: Different combinations of input data (various combinations of cLFC at days 7, 11, 15 and 21) and their performance in a Random Forest supervised classifier.

Supplementary Data S25: Calculation of the weighting which can be applied to the DDX3X SGE data within the ACMG clinical variant classification according to guidelines described by Brnich *et al.* 2019.

Supplementary Data S26: *DDX3X* missense variants in 90,279 independent cancer samples obtained from cBioPortal.

Supplementary Data S27: dNdScv results (observed/expected calculation of DDX3X variants) in individual cancers split by sex.

Supplementary Data S28: dNdScv results for the following groups: Medulloblastoma, non-medulloblastoma DDX3X driver cancers, all DDX3X driver cancers non-DDX3X driver cancers. DDX3X driver/non-driver groups derived from Supplementary data S22.

Supplementary Movie Legends:

Supplementary movie SM1: Alphafold2 DDX3X structure, amino acids coloured according to the modal missense functional class at each residue.

Supplementary movie SM1: Alphafold2 DDX3X structure, amino acids coloured according to the SGE functional class when this amino acid is deleted
