# Supplementary material for: Saturation genome editing of DDX3X clarifies pathogenicity of germline and somatic variation
Source: Nat Commun. 2023 Dec 6;14:7702. doi: 10.1038/s41467-023-43041-4 (PMC10700591; doi:10.1038/s41467-023-43041-4)
Supplement: Supplementary file 7 — Reporting Summary [file 41467_2023_43041_MOESM7_ESM.pdf]

nature portfolio

Corresponding author(s): Matthew Hurles

Last updated by author(s): Sep 18, 2023

Reporting Summary

Nature Portfolio wishes to improve the reproducibility of the work that we publish. This form provides structure for consistency and transparency in reporting. For further information on Nature Portfolio policies, see our Editorial Policies and the Editorial Policy Checklist.

Statistics

For all statistical analyses, confirm that the following items are present in the figure legend, table legend, main text, or Methods section.

n/a	Confirmed	
☐	☒	The exact sample size (*n*) for each experimental group/condition, given as a discrete number and unit of measurement
☐	☒	A statement on whether measurements were taken from distinct samples or whether the same sample was measured repeatedly
☐	☒	The statistical test(s) used AND whether they are one- or two-sided<br>*Only common tests should be described solely by name; describe more complex techniques in the Methods section.*
☐	☒	A description of all covariates tested
☐	☒	A description of any assumptions or corrections, such as tests of normality and adjustment for multiple comparisons
☐	☒	A full description of the statistical parameters including central tendency (e.g. means) or other basic estimates (e.g. regression coefficient) AND variation (e.g. standard deviation) or associated estimates of uncertainty (e.g. confidence intervals)
☐	☒	For null hypothesis testing, the test statistic (e.g. *F*, *t*, *r*) with confidence intervals, effect sizes, degrees of freedom and *P* value noted<br>*Give P values as exact values whenever suitable.*
☒	☐	For Bayesian analysis, information on the choice of priors and Markov chain Monte Carlo settings
☒	☐	For hierarchical and complex designs, identification of the appropriate level for tests and full reporting of outcomes
☐	☒	Estimates of effect sizes (e.g. Cohen's *d*, Pearson's *r*), indicating how they were calculated

Our web collection on statistics for biologists contains articles on many of the points above.

Software and code

Policy information about availability of computer code

Data collection	No software was used during the data collection.
Data analysis	These free software were used in the data analysis:<br>1) Python 2/3<br>2) R 4.0.2<br>3) Trim-galore v0.6.4_dev<br>4) Tagdust v2.33<br>5) BLASTP<br>6) VaLiAnT v1.0.0 (https://github.com/cancerit/VaLiAnT)<br>7) FlowJo v10.6.1<br>8) bcftools 1.9, Using htslib 1.9<br>9) DESeq2 v1.30.1<br><br>The custom codes for the data analysis can be found at https://github.com/HurlesGroupSanger/Saturation_Genome_Editing

For manuscripts utilizing custom algorithms or software that are central to the research but not yet described in published literature, software must be made available to editors and reviewers. We strongly encourage code deposition in a community repository (e.g. GitHub). See the Nature Portfolio guidelines for submitting code & software for further information.

Data

Policy information about availability of data

All manuscripts must include a data availability statement. This statement should provide the following information, where applicable:

- Accession codes, unique identifiers, or web links for publicly available datasets
- A description of any restrictions on data availability
- For clinical datasets or third party data, please ensure that the statement adheres to our policy

The DNA sequencing data of HAP1 cells isolated from the SGE experiment at days 4, 7, 11, 15 and 21 generated in this study were deposited to ENA with accession number PRJEB52929. The analysed data were deposited in MaveDB with accession number urn:mavedb:00000658.
The data used for the analyses described in this manuscript were obtained from the GTEx Portal on 02/02/23. The results used in the analysis are in part based upon data generated by the TCGA Research Network: https://www.cancer.gov/tcga. In addition, variants reported in ClinVar (4th December 2020), DECIPHER (4th December 2020), Genomics England 100,000 genomes study (21st January 2021), gnomAD v2.1.1 and gnomAD v3.1 were used in the analysis. Source data are provided with this paper.

Human research participants

Policy information about studies involving human research participants and Sex and Gender in Research.

Reporting on sex and gender	N/A
Population characteristics	N/A
Recruitment	N/A
Ethics oversight	N/A

Note that full information on the approval of the study protocol must also be provided in the manuscript.

Field-specific reporting

Please select the one below that is the best fit for your research. If you are not sure, read the appropriate sections before making your selection.

☒ Life sciences ☐ Behavioural & social sciences ☐ Ecological, evolutionary & environmental sciences

For a reference copy of the document with all sections, see nature.com/documents/nr-reporting-summary-flat.pdf

Life sciences study design

All studies must disclose on these points even when the disclosure is negative.

Sample size	We intended to assay all the designed variants in the SGE experiment. We did not perform a calculation of the sample size required for this experiment. To avoid insufficient sampling, the cells were maintained with at least 10,000X library coverage for every passage.
Data exclusions	During SGE analysis, we exclude the variants which have a low read count in all 15 samples/replicates (triplicates of 5 timepoints). Sequences with a total read count less than or equal to 10 were removed from the analysis.
Replication	We performed 3 independent transfections (3 biological replicates) for each SGE experiment. The replicates were reproducible, except for, Exon3 sg1 Day4 Rep1, Exon9 sg1 Day4 Rep2 and Exon9 sg2 Day4 Rep3. These samples either formed a distinct cluster in the unsupervised clustering analysis or had a high correlation to the plasmid data, indicating potential contamination from the HDR plasmid library. These bad replicates were excluded from the analysis.
Randomization	For each of the SGE assays, hundreds of variants were tested in multiplex. The variants were randomly knocked in to the DDX3X gene locus by homology directed repair (HDR) mediated by the CRISPR-Cas9 system.
Blinding	The caculation of LFC, LFC-trend and SGE functional class for each variant were performed without knowledge of their clinical pathogenicity status.

Reporting for specific materials, systems and methods

We require information from authors about some types of materials, experimental systems and methods used in many studies. Here, indicate whether each material, system or method listed is relevant to your study. If you are not sure if a list item applies to your research, read the appropriate section before selecting a response.

Materials & experimental systems

n/a	Involved in the study
☒	☐ Antibodies
☐	☒ Eukaryotic cell lines
☒	☐ Palaeontology and archaeology
☒	☐ Animals and other organisms
☒	☐ Clinical data
☒	☐ Dual use research of concern

Methods

n/a	Involved in the study
☒	☐ ChIP-seq
☐	☒ Flow cytometry
☒	☐ MRI-based neuroimaging

Eukaryotic cell lines

Policy information about cell lines and Sex and Gender in Research

Cell line source(s)	HAP1 LIG4 knock-out cells were purchased from Horizon Discovery product-ID HZGHC000759c006.
Authentication	The HAP1 cell was authenticated by karyotype. 10bp deletion at the LIG4 locus was validated by Sanger sequencing.
Mycoplasma contamination	Cell lines were tested negative for mycoplasma.
Commonly misidentified lines (See ICLAC register)	No commonly misidentified cell lines were used.

Flow Cytometry

Plots

Confirm that:

☒ The axis labels state the marker and fluorochrome used (e.g. CD4-FITC).

☒ The axis scales are clearly visible. Include numbers along axes only for bottom left plot of group (a 'group' is an analysis of identical markers).

☒ All plots are contour plots with outliers or pseudocolor plots.

☒ A numerical value for number of cells or percentage (with statistics) is provided.

Methodology

Sample preparation	For FACS analysis on GFP percentage, the pMAX GFP co-transfected HAP1 and the non-transfected HAP1 cell were trypsinized. The enzyme was inactivated by the addition of HAP1 culture medium (IMDM, 10% FBS). DAPI was added to the cell suspension at 1ug/mL final concentration. The cells were filtered through a 40um cell strainer before FACS.<br><br>For the ploidy assay, the sample preparation was described in the Method section. In short, the nocodazole treated cells were trypsinized and fixed with cold 80% ethanol. The cells were then stained with DAPI at 1ug/mL final concentration.
Instrument	BD Fortessa and Cytoflex
Software	FlowJo v10.6.1
Cell population abundance	GFP+ cell: 44.2%<br>Day0 SGE diploid cell : 4.02%<br>Day23 SGE diploid cell: 23.9%
Gating strategy	GFP gating: Cell> singlets > viable > GFP positive<br>Ploidy gating: Cell > singlet > diploid (2n)

☒ Tick this box to confirm that a figure exemplifying the gating strategy is provided in the Supplementary Information.
